# Oxidoreductases and metal cofactors in the functioning of the earth

**DOI:** 10.1042/EBC20230012

**Published:** 2023-08-11

**Authors:** Bruno Hay Mele, Maria Monticelli, Serena Leone, Deborah Bastoni, Bernardo Barosa, Martina Cascone, Flavia Migliaccio, Francesco Montemagno, Annarita Ricciardelli, Luca Tonietti, Alessandra Rotundi, Angelina Cordone, Donato Giovannelli

**Affiliations:** 1 Department of Biology, University of Naples Federico II, Naples, Italy; 2 National Research Council - Institute of Biomolecular Chemistry - CNR-ICB, Pozzuoli, Italy; 3 Dipartimento di Biologia ed Evoluzione degli Organismi Marini, Stazione Zoologica Anton. Dohrn, Napoli, Italy; 4 Department of Science and Technology, University of Naples Parthenope, Naples, Italy; 5 National Research Council - Institute of Marine Biological Resources and Biotechnologies - CNR-IRBIM, Ancona, Italy; 6 Department of Marine and Coastal Science, Rutgers University, New Brunswick, NJ, U.S.A.; 7 Marine Chemistry and Geochemistry Department - Woods Hole Oceanographic Institution, MA, U.S.A.; 8 Earth-Life Science Institute, Tokyo Institute of Technology, Tokyo, Japan

**Keywords:** biogeochemistry, ligands, metabolism, metalloproteins, organometallic compounds, Redox reactions

## Abstract

Life sustains itself using energy generated by thermodynamic disequilibria, commonly existing as redox disequilibria. Metals are significant players in controlling redox reactions, as they are essential components of the engine that life uses to tap into the thermodynamic disequilibria necessary for metabolism. The number of proteins that evolved to catalyze redox reactions is extraordinary, as is the diversification level of metal cofactors and catalytic domain structures involved. Notwithstanding the importance of the topic, the relationship between metals and the redox reactions they are involved in has been poorly explored. This work reviews the structure and function of different prokaryotic organometallic–protein complexes, highlighting their pivotal role in controlling biogeochemistry. We focus on a specific subset of metal-containing oxidoreductases (EC1 or EC7.1), which are directly involved in biogeochemical cycles, i.e., at least one substrate or product is a small inorganic molecule that is or can be exchanged with the environment. Based on these inclusion criteria, we select and report 59 metalloenzymes, describing the organometallic structure of their active sites, the redox reactions in which they are involved, and their biogeochemical roles.

## Introduction

**Figure 1:**
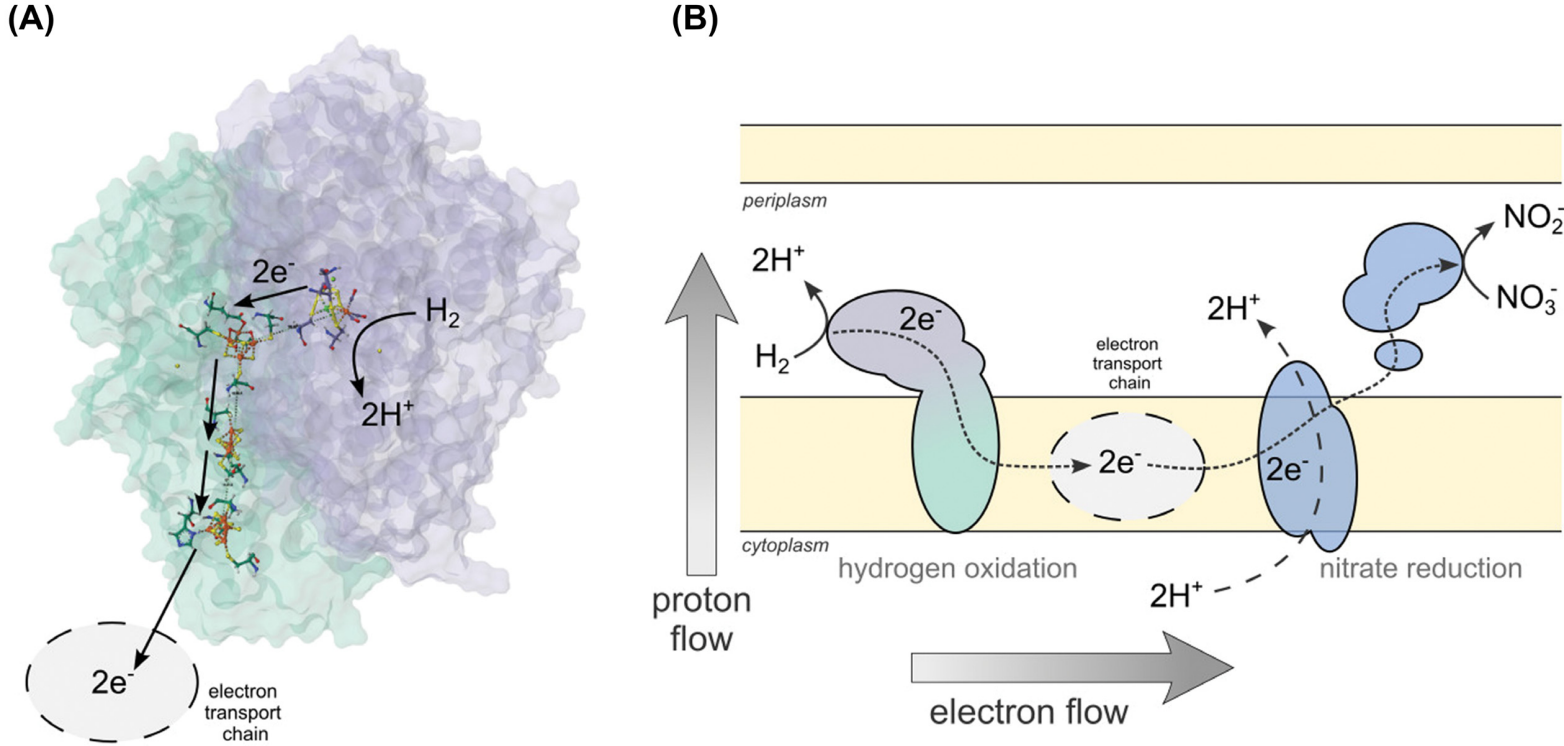
Oxidoreductase proteins and electron stranspor chain used by life to capture redox disequilibrium. (**A**) The heterodimeric structure of the periplasmic [NiFe]-hydrogenase from *Desulfovibrio vulgaris* (hyd, 5XLF) showing the [NiFe] catalytic center and the iron-sulfur clusters responsible for the electron transport. (**B**) Cellular model of the coupling between the periplasmic membrane-bound [NiFe]-hydrogenase Hyd and the periplasmic molybdopterin-containing nitrate reductase Nap in *Thermovibrio ammonificans* showing the decoupling between electrons and protons across the membrane (adapted from [83]).

Life is fundamentally electric [1]. The thermodynamic disequilibria present in the environment as geochemical gradients are exploited by life to drive its metabolic reactions. The two energy sources used by life, chemical for chemotrophs and light for phototrophs, are always linked or converted to redox disequilibria. Thus life’s need for thermodynamic disequilibrium is ultimately a requirement for redox chemistry imbalance. Thermodynamically favorable redox reactions (e.g*.*, glucose oxidation coupled to oxygen respiration) are chopped by life into sub reactions decoupling the flow of electrons and protons through the electron transport chain and the cell membrane to create a chemiosmotic gradient (Figure 1). This separation effectively converts a scalar (directionless) redox chemical reaction into a vectorial (gradient-forming) process, producing chemical and mechanical work. In a sense, life has solved the need for energy to drive biochemical reactions anticipating Alessandro Volta’s battery by nearly four billion years [2].

**Figure 2:**
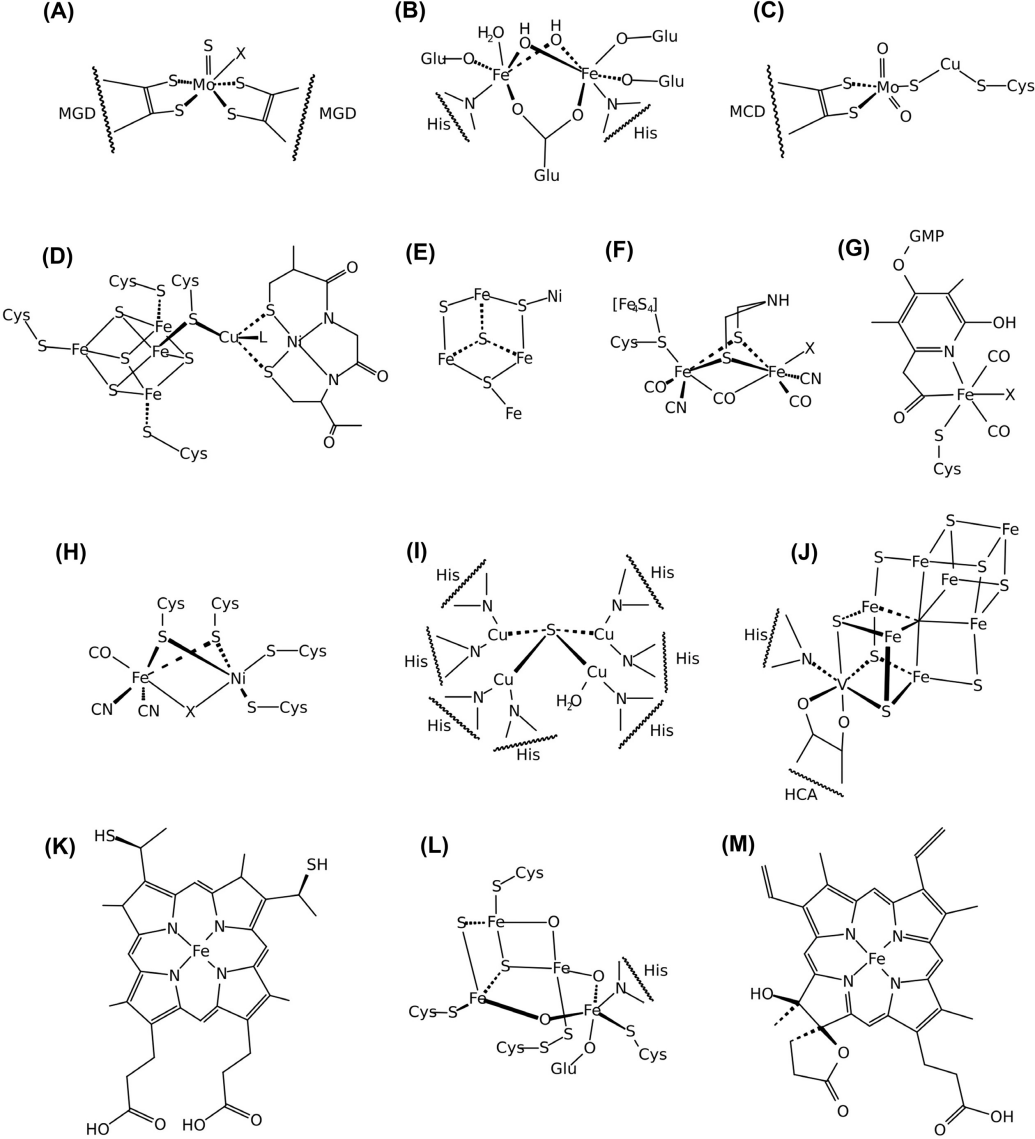
Chemical structures of biogeochemically relevant metal-containing cofactors from prokaryotic oxidoreductases. (**A**) the Mo-containing catalytic site of Formate dehydrogenase (*fdhF*, 1FDO; shown in Figure 3D); (**B**) the FeO cluster of the soluble methane monooxygenase hydroxylase (*mmoX*,1MHY); (**C**) the Mo-Cu-containing cluster in the active site of CO dehydrogenase (*coxL*, 1ZXI); (**D**) the Ni-Fe-Cu center (cluster A) and (**E**) the Fe-[NiFe3S4] cofactor (cluster C) of the anaerobic carbon monoxide dehydrogenase (*codh*, 1MJG); (**F**) the catalytic centers of the [FeFe]-hydrogenase (*hydA*, 6N59; shown in Figure 3A), [Fe]-hydrogenase (**G**) (*hmd*, 6HAV; shown in Figure 3C) and [NiFe]-hydrogenase (**H**) (*hydB*, 5XLF; shown in Figure 3B); (**I**) the [Cu_4_S] cluster of the nitrous oxide reductase (*nosZ*, 1FWX); (**J**) the FeVco cofactor of the V-containing nitrogenase (*vnfD*, 5N6Y; shown in Figure 3G); (**K**) the Heme C contained in several oxidoreductases (*hzsA, hdh, nrfA, nirS, hao, tsdA* [5C2V, 6HIF, 2J7A, 6TSI, 1FGJ, 4V2K]); (**L**) the hybrid cluster from the the Hybrid Cluster Protein from Desulfovibrio vulgaris (*hcp*, 1E1D); (**M**) the Cis-heme hydroxychlorin gamma-spirolactone (*cydA* and *appC*, 6RKO and 7OY2).

Biology has evolved proteins that act stepwise to control redox reactions, transferring electrons across redox states between the opening donor and the ultimate acceptor. These proteins, called oxidoreductases (classified under the Enzyme Commission classes 1 and 7.1), are overwhelmingly metal-containing. To precisely and efficiently transfer electrons to and from a wide range of molecules, they finely tune their conjugated metals' midpoint electric potential by controlling the coordination sphere, geometry, and accessibility of the active site [3]. Elements incorporated in the oxidoreductases’ catalytic centers include transition metals such as Fe, Mo, W, Zn, Cu, V, Mn, Ni, Mg, Co and Se and non-metals like S, coordinated either directly or through organometallic structures in the active center [4] (Figure 2). Despite the critical role of metalloproteins in biology, our understanding of the diversity of elements and structures they use is still limited. For example, recent work has demonstrated that lanthanides, a group of elements previously believed to be inert for life, are used by an enzyme catalyzing a key step in the aerobic respiration of methane [5].

## The functioning of our planet: a focus on biogeochemistry

Redox couples are recycled on a planetary scale by coupled geological and biological processes happening at diverse spatial and temporal scales. Within biology, redox cycling of key macromolecule-building elements (e.g*.*, carbon, hydrogen, nitrogen, oxygen, and sulfur, also known as CHNOS elements) is primarily carried out by microorganisms inhabiting diverse ecosystems [6]. We did not consider phosphorus, an essential building block in biochemistry, since its biogeochemical cycle is governed by Lewis acid–base chemistry rather than redox chemistry [7].

Most key reactions that control biogeochemistry are carried out by a small set of microbial-encoded proteins containing a redox-sensitive transition metal as core catalytic center [8]. Life can exploit thermodynamic disequilibria present in natural systems using these enzymes whenever the kinetics of the abiotic reactions is slow enough or the activation energy required is big enough for life to outcompete it [9].

Here we discuss the diversity of metal-containing catalytic structures in essential biogeochemical redox proteins and their importance in our planet's functioning. While all enzymes participating in a given metabolic pathway are essential, and all are critical in biogeochemistry regardless of the metabolism itself, this review focused on a small subset of enzymes selected following these criteria:

1. They are exclusively oxidoreductases (EC1 or EC7.1), given the dependence of life on redox chemistry.
2. They are metal-containing proteins (metalloproteins). Metals often occur in multiple subunits participating in the redox reaction and passing electrons within the enzyme complex. Here, we have considered only oxidoreductases in which the metal directly participates in the primary redox reactions.
3. They are biogeochemically relevant, i.e., they catalyze a reaction where the substrate/product is a small inorganic molecule that is (or can be) directly exchanged with the environment. Enzymes interacting with molecules like CO_2_, CO, H_2_, NO_3_^−^, NH_4_^+^, SO_4_^2−^, H_2_S, and many other compounds fall in this category. Methane (CH_4_), considered an organic molecule, is included in this work’s list of valid biogeochemical compounds.

These criteria exclude all the enzymes that, while fundamental for the functioning of metabolism, interact with metabolic intermediates and all the key enzymes that do not deal with redox reactions-for example, the key enzyme for the Calvin–Benson–Bassam cycle, Rubisco (EC 4.1.1.39), and many essential genes involved in carbon fixation. In addition, metal-containing oxidoreductase complexes without a metal in the active site are excluded. An example in this category is the flavocytochrome c sulfide dehydrogenase (EC 1.8.5.4), responsible for the reversible conversion of sulfide to elemental sulfur in several sulfide oxidizers and anoxygenic phototrophs. While the heterodimer contains two heme cofactors (making it an iron-containing metalloprotein) and interacts with both H_2_S and elemental sulfur, the active site of the catalytic subunit does not contain any metal. It uses instead two flavin-adenine dinucleotide (FAD) cofactors [10]. Under these criteria, the number of biogeochemically-relevant metal-containing oxidoreductases involved in key steps of the CHNOS cycles is reduced to 59 (Table 1 and Supplementary Figure S1). These effectively control the biogeochemistry at the interface between the geosphere and biosphere and are more likely to be influenced by the environmental availability of their metal cofactor [11].

**Table 1:** List of biogeochemically relevant metals containing oxidoreductases controlling the major CHNOS cycles

Cycle	Pathway	Step	Uniprot	Protein name	Gene	EC	KO	PFAM	Metal in the active center	Organometallic structure [PDB ligand accession]	PDB	Representative organism
Carbon	Aerobic methane oxidation	Oxygenation of methane to methanol	G1UBD1	Particulate methane monooxygenase α subunit	*pmoB1, pmoB2*	1.14.18.3	K10944	PF04744	Cu	Cu (II)-Cu (II) [CUA]	3RGB	*Methylococcus capsulatus*
			P27354	(soluble) Methane monooxygenase component A β chain	*mmoX*	1.14.13.25	K16157	PF02332	Fe	2 Fe (III) [FE]	1MHY	*Methylosinus trichosporium*
	Carbon fixation	CO_2_ reduction to formate	P07658	Formate dehydrogenase H	*fdhF*	1.17.98.4	K22015	PF04879	Mo	Mo (VI) [MO] molybdenum-bis (molybdopterin guanine dinucleotide) [MGD]	1FDO	*Escherichia coli*
		CO_2_ reduction to formylmethanofuran	O74030	Tungsten formylmethanofuran dehydrogenase subunit fwdA	*fwdA*	1.2.7.12	K00200	PF00384	W	W (VI) [W], molybdenum-bis (molybdopterin guanine dinucleotide) [MGD]	5T5I	*Methanothermo- bacter wolfeii*
		Oxidation of CO to CO_2_	P19920	(aerobic) Carbon monoxide dehydrogenase medium chain	*coxL*	1.2.5.3	K03520	PF02738, PF20256	CuMo	Cu (I) -S- Mo (VI) (= O) OH Cluster [CUM]	1ZXI	*Oligotropha carboxidovorans*
		Reduction of CO_2_ to CO	P27988	(anaerobic) Carbon monoxide dehydrogenase/ acetyl-CoA synthase subunit alpha	*codh*	1.2.7.4	K00192	PF03063	CuNi/Ni	Fe(4)-Ni(1)-S (4) Cluster [XCC] + Cu Ion [CU1]	1MJG	*Moorella thermoacetica*
Hydrogen	Hydrogen oxidation	F420 reduction	D9PYF9	(NiFe) F420-reducing hydrogenase, subunit α	*frhA*	1.12.98.1	K00440	PF00374	NiFe	Formyl [bis(hydrocyanato -1kappaC)] Fe-Ni [NFU]	4OMF	*Methanthermo- bacter marburgensis str. Marburg*
		H_2_-respiration	Q58194	5,10-methenyl tetrahydromethanopterin hydrogenase	*hmd*	1.12.98.2	K13942	PF03201	Fe	Fe (II) [FE2] coordinated by 5'-O-[(S)-hydroxy {[2-hydroxy-3,5- dimethyl-6-(2- oxoethyl)pyridin- 4-yl]oxy} phosphoryl] guanosine [I2C]	3F47	*Methancaldo- coccus jannaschii*
			P21852	Periplasmic [NiFe] hydrogenase large subunit	*hydB*	1.12.2.1	K00437	PF00374	NiFe/NiFeSe	Ni-Fe oxidized active center [NFV] or NiFeSe	5XLF	*Desulfovibrio vulgaris*
	Hydrogen production	H_2_-production	P29166	(FeFe) Iron hydrogenase 1	*hydA*	1.12.7.2	K00533	PF02906	Fe	dicarbonyl [bis(cyanide- kappaC)]-mu- (imin dimethanethiol- atato-1kappaS: 2kappaS)-mu -(oxomethylidene) diiron (II) [402]	6N59	*Clostridium pasteurianum*
		H_2_-production/Mrp Antiporter	Q8U0Z6	(NiFe) Membrane-bound hydrogenase subunit α	*mbhL*	1.12.7.2	K18016	PF1434	NiFe	Formyl [bis(hydrocyanato- 1kappaC)]Fe-Ni [NFU]	6CFW	*Pyrococcus furiosus*
Nitrogen	Anammox	From ammonia to hydrazine	Q1Q0T3	Hydrazine synthase subunit γ	*hzsA*	1.7.2.7	K20932	PF18582	Fe	HEME C [HEC]	5C2V	*Candidatus Kuenenia stuttgartiensis*
		From hydrazine to nitrogen	Q1PW30	Hydrazine dehydrogenase	*hdh*	1.7.2.8	K20935	PF13447	Fe	HEME C [HEC]	6HIF	*Candidatus Kuenenia stuttgartiensis*
	Assimilatory nitrate reduction	From nitrate to nitrite	P73448	Nitrate reductase	*narB*	1.7.7.2	K00367	PF00355	Mo	Mo (VI [MO] + 2 molybdenum-bis (molybdopterin guanine dinucleotide) [MGD]	AF-P73448-F1	*Synechocystis* sp. PCC 6803
		From ammonia to nitrite	P9WJ03	Ferredoxin-nitrite reductase	*nirA*	1.7.7.1	K00366	PF01077, PF03460	Fe	Siroheme [SRM]	1ZJ8	*Mycobacterium tuberculosis H37Rv*
	Dissimilatory nitrite reduction	From ammonia to nitrite	Q72EF3	Cytochrome *c* nitrite reductase subunit NrfA	*nrfA*	1.7.2.2	K03385	PF02335	Fe	HEME C [HEC]	2J7A	*Desulfovibrio vulgaris str. Hildenborough*
	Denitrification	From nitrate to nitrite	P09152	Respiratory nitrate reductase 1 α chain	*narG*	1.7.5.1	K00370	PF00384, PF01568	Mo	Mo (VI) [MO] + 2 PO4-(2-amino-4- oxo-3,4,5,6,- tetrahydro-pteridic- 6-YL)-2-hydroxy- 3,4-dimercapto- butenyl ester guamylate [MD1]	1Y4Z	*Escherichia coli*
			P81186	Periplasmic nitrate reductase	*napA*	1.9.6.1	K02567	PF04879, PF00384, PF01568	Mo	Mo (VI) [MO] + 2 molybdenum-bis (molybdopterin guanine dinucleotide) [MGD]	2JIM	*Desulfovibrio desulfuricans*
		From Nitric oxide to nitrite	E8PLV7	Copper-containing nitrite reductase	*nirK*	1.7.2.1	K00368	PF00394, PF00732	Cu	Cu (II) [CU]	6HBE	*Thermus scotoductus*
			P24474	Nitrite reductase	*nirS*	1.7.2.1	K15864	PF02239, PF13442	Fe	HEME C [HEC]	6TSI	*Pseudomonas aeruginsa*
		From nitrite to nitrate	P49050	Nitrate reductase [NADPH]	*nasA*	1.7.7.2	K00372	PF04879, PF00384, PF01568	Mo	(Molybdopterin -S,S)-dioxo- thio-Mo (IV) [MTV]	2BIH	*Ogataea angusta*
		From nitrogen to nitrous oxide	Q51705	Nitrous-oxide reductase	*nosZ*	1.7.2.4	K00376	PF00116, PF18764, PF18793	Cu	Cu4S [CUZ]	1FWX	*Paracoccus denitrificans*
		From nitrous oxide to nitric oxide	B3Y963	Nitric oxide reductase	*norB*	1.7.2.5	K04561	PF00115	Fe	Protoporph- yrin IX containing Fe [HEM]	3AYF	*Geobacillus stearothermophilus*
	Nitrification	From ammonia to hydroxylamine	Q04508	(Cupredoxin) Ammonia monooxygenase beta subunit	*amoB1; amoB2*	1.14.99.39	K10944	PF02461	Cu	Cu (II) [CU]	AF-Q04508-F1	*Nitrosomonas europaea*
			P31101	Hydroxylamine reductase	*hcp*	1.7.99.1	K05601	PF03063	Fe	Iron/Sulfur/ Oxygen Hybrid Cluster [FSO]	1E1D	*Desulfovibrio vulgaris*
		From ammonia to nitrite	P08201	Nitrite reductase (NADH) large subunit	*nirB*	1.7.1.15	K00363	PF04324, PF01077, PF03460, PF07992, PF18267	Fe	–	AF-P08201-F1	*Escherichia coli (strain K12)*
		From hydroxilamine to nitrite	Q50925	Hydroxylamine oxidoreductase	*hao*	1.7.2.6	K10535	PF13447	Fe	HEME C [HEC]	1FGJ	*Nitrosomonas europaea*
		From nitrite to nitrate	Q1PZD8	Nitrite oxidoreductase subunit A	*nrxA*	1.7.99.-	K00370	PF09459	Mo	Mo (VI) [MO] + 2 PO4-(2-amino-4 -oxo-3,4,5,6,- tetrahydro-pteridic-6 -YL)-2-hydroxy-3, 4-dimercapto-butenyl ester guamylate [MD1]	7B04	*Candidatus Kuenenia stuttgartiensis*
	Nitrogen fixation	From nitrogen to ammonia	-^a^	Nitrogenase iron–iron protein	*anfD*	1.18.6.1	K00531	PF00148	Fe	FeFeco^a^	8OIE ^a^	*Azotobacter Vinelandi*
			P07328	Nitrogenase molybdenum–iron protein alpha chain	*nifD*	1.18.6.1	K02586	PF00148	Mo	FeMoco [ICS]	3U7Q	*Azotobacter Vinelandi*
			P16855	Nitrogenase vanadium–iron protein α chain	*vnfD*	1.18.6.1	K22896	PF00148	V	FeVco [8P8]	5N6Y	*Azotobacter Vinelandi*
Oxygen	Oxygen radicals detoxification	Hydrogen peroxide detoxification	Q3JNW6	Catalase-peroxidase	*katG*	1.11.1.21	K03782	PF00141	Fe	Protoporph- yrin IX Containing FE [HEM]	5SW4	*Burkholderia pseudomallei*
		Oxygen detoxification	P0ABE5	Superoxide oxidase CybB	*cybB*	1.10.3.17	K12262	PF01292	Fe	Protoporph- yrin IX Containing FE [HEM]	5OC0	*Escherichia coli*
		Superoxide detoxification	P80734	Superoxide dismutase [Ni]	*sodN*	1.15.1.1	K00518	PF09055	Ni	Ni (II) [NI]	1Q0G	*Streptomyces seoulensis*
			P00446	Superoxide dismutase [Cu-Zn]	*sodC*	1.15.1.1	K04565	PF00080	Cu	Cu (II) [CU]	1BZO	*Photobacterium leiognathi*
			Q9RUV2	Superoxide dismutase [Mn]	*sodA*	1.15.1.1	K04564	PF02777, PF00082	Fe/Mn	Fe (III) [FE]/Mn (II) [MN]	1Y67, 3KKY	*Deincoccus radiodurans*
			P82385	Superoxide reductase	*sorA*	1.15.1.2	K05919	PF06397, PF01880	Fe	Fe (III) [FE]	1DQI	*Desulfovibrio desulfuricans*
	Oxygen respiration	Oxidative phosphorylation	D9IA44	*Cbb3*-type cytochrome *c* oxidase (subunit II)	*ccoN*	7.1.1.9	K00404	PF00115	Cu	Cu (II) [CU] + Protoporphyrin IX [HEM]	5DJQ	*Stutzerimonas stutzeri*
			P34956	Cytochrome ba quinol oxidase subunit 1	*qoxB*	7.1.1.5	K02827	PF00115	Cu	Cu (II) [CU] + Heme-A [HEA]	6KOB	*Bacillus subtilis*
			P0ABJ9	Cytochrome bd-I ubiquinol oxidase subunit 1	*cydA*	7.1.1.7	K00425	PF01654	Fe	Cis-heme D hydroxychlorin gamma- spirolactone [HDD]	6RKO	*Escherichia coli*
			P24244	Putative cytochrome bd-II ubiquinol oxidase subunit AppX	*appC*	7.1.1.7	K00425	PF01654	Fe	Cis-heme D hydroxychlorin gamma- spirolactone [HDD]	7OY2	*Escherichia coli*
			P0ABJ6	Cytochrome bo(3) ubiquinol oxidase subunit 4	*cyoB*	7.1.1.3	K02298	PF00115	Cu	Cu (II) [CU] + HEME-O [HEO]	7N9Z	*Escherichia coli*
			P98005	Cytochrome-*c* oxidase polypeptide I + III	*ctaD*	7.1.1.9	K02274	PF00115	Cu	Cu (II) [CU] + Heme-AS [HAS]	2YEV	*Thermus thermophilus*
	Oxygenic photosynthesis	Water oxidation to oxygen	P0A444	Photosystem II protein D1 1	*psbA1*	1.10.3.9	K02703	PF00124	Mn	Oxygen evolving system [OEC]	3KZI	*Thermosynecho- coccus elongatus*
Sulfur	Aerobic sulfur disproportionation	From S-sulfanylglu- tathione to glutathione + sulfite	A5VWI3	Sulfur dioxygenases	*sdoA*	1.13.11.18	–	PF00753	Fe	Fe (III) [FE]	4YSK	*Pseudomonas putida*
		Catalyzes the simultaneous oxidation and reduction of elemental sulfur in the presence of oxygen	P29082	Sulfur oxygenase/reductase	*sor*	1.13.11.55	K16952	PF07682	Fe	Fe (III) [FE]	2CB2	*Acidianus ambivalens*
	Assimilatory sulfate reduction	Reduction of sulfite to sulfide	A0A920E3E6	Assimilatory sulfite reductase (ferredoxin)	*sir*	1.8.7.1	K00392	PF03460, PF01077	Fe	SIROHEME [SRM]	–	*Synechococcus* sp. PCC7942
			P17846	Sulfite reductase [NADPH] hemoprotein beta-component	*cysL*	1.8.1.2	K00381	PF01077, PF03460	Fe	SIROHEME [SRM]	1AOP	*Escherichia coli*
	DMSO reduction	Catalyzes the conversion of DMSO to dimethyl sulfide	Q57366	Dimethyl sulfoxide/trimethylamine N-oxide reductase	*dmsA*	1.8.5.3	K07306	PF04879, PF01568, PF00384	Mo	Mo (VI [MO] + 2 molybdenum-bis (molybdopterin guanine dinucleotide) [MGD]	1EU1	*Rhodobacter sphaeroides*
		DMSO reduction	Q8GPG4	Dimethylsulfide dehydrogenase subunit α	*ddhA*	1.8.2.4	K16964	PF00384, PF01568	Mo	Mo (VI [MO] + 2 molybdenum-bis (molybdopterin guanine dinucleotide) [MGD]	AF-Q8GPG4-F1	*Rhodovulum sulfidophilum*
	Sulfate reduction	Catalyzes the reduction of sulfite to sulfide	Q59109	Sulfite reductase, dissimilatory-type subunit α	*dsrA*	1.8.99.5	K11180	PF03460, PF01077	Fe	Siroheme [SRM]	3MM5	*Archeoglobus fulgidus*
	Sulfite oxidation	Sulfite oxidation to sulfate	D3RNN8	Sulfite dehydrogenase subunit A	*soeA*	1.8.5.6	K21307	PF04879, PF00384, PF01568	Mo	Mo (VI [MO] + 2 molybdenum-bis (molybdopterin guanine dinucleotide) [MGD]	AF-D3RNN8-F1	*Allochromatium vinosum*
	Sulfur disproportionation	From sulfite to sulfate	Q9LA16	Sulfite: cytochrome c oxidoreductase subunit A	*sorA*	1.8.2.1	K05301	PF00174, PF03404	Mo	(molybdopte- rin- S,S)-oxo -Mo [MSS]	2BPB	*Starkeya novella*
	Sulfur reduction	Catalyzes the cytoplasmic production of hydrogen sulfide in the presence of elemental sulfur	–	Sulfhydrog- enase	*shyB*	1.12.98.4	K17995, K17996	PF17179, PF00175, PF10418	Fe	–	–	*Pyrococcus furiosus*
		Sulfur reduction	Q8NKK1	Sulfur reductase molybdopterin subunit	*sreA*	1.97.1.3/1.12.98.4	K17219	PF04879, PF01568, PF00384	Mo	Mo (VI) [MO] + 2 molybdenum-bis (molybdopterin guanine dinucleotide) [MGD]	AF-Q8NKK1-F1	*Acidianus ambivalens*
	Thiosulfate oxidation	From thiosulfate to sulfate	O07819	Sulfur-oxidation complex	*soxCD*	1.8.2.6	K17225	PF00174, PF03404	MoCo	Mo (IV) oxide [2MO] + Co (II) [CO]	2XTS	*Paracoccus pantotrophus*
		From thiosulfate to tetrathionate	D3RVD4	Thiosulfate dehydrogenase	*tsdA*	1.8.2.2	K19713	PF13442	Fe	HEME C [HEC]	4V2K	*Allochromatium vinosum*
	Thiosulfate reduction	From thiosulfate to hydrogen sulfide	Q72LA6	Polysulfide reductase chain A	*phsA/psrA*	1.8.5.5	K08352	PF04879, PF00384, PF01568	Mo	Mo (VI) [MO] + 2 molybdenum-bis (molybdopterin guanine dinucleotide) [MGD]	2VPX	*Thermus thermophilus*
	Sulfite reduction	Reduces sulfite to sulfide	Q58280	Coenzyme F420-dependent sulfite reductase	*fsr*	1.8.98.3	K21816	PF00037, PF04432, PF04422, PF01077, PF03460	Fe	SIROHEME [SRM]	7NP8	*Methanocaldo- coccus jannaschii*

PDB and UniProt accessions are reported for each gene, together with Ligand ID; AlphafoldDB codes are reported when a crystallographic structure is unavailable. For enzymes known to be cambialistic (i.e*.*, accept alternative metals in the active site) in experimental setups, the alternative metals are reported separated by a “/”. An extended version of the table reporting all the other cofactors present in the catalytic subunit of the enzyme is available as supplementary online material and published on a permanent archive with doi: 10.5281/zenodo.7934782. *a* - the structure, organometallic structure and Uniprot accession number for the Fe nitrogenase is on hold at the time of writing and awaiting release.

## Carbon cycle

At the most fundamental level, life is carbon-based. Hence, life plays a vital role in mediating the biogeochemical cycles of carbon on earth’s surface [12,13]. Inorganic carbon is a building block for assembling complex C molecules [12] through autotrophy-based metabolic strategies. At the same time, CH_4_ can be oxidized to yield energy for cellular growth and maintenance, ultimately releasing CO_2_ [14,15]. While oxidoreductases are involved in the pathways responsible for the uptake and release of inorganic carbon compounds, few are metal-containing oxidoreductases. The KEGG database lists 66 enzymatic classes involved in carbon fixation pathways, of which 21 are classified as oxidoreductases. However, only three (5% of all enzymatic classes involved in carbon fixation pathways) fall within our definition (Table 1). Other carbon-related metabolisms important at the biogeochemical level are methanogenesis, aerobic and anaerobic methane oxidation, and carbon monoxide utilization. KEGG lists 33 enzymatic classes involved in these pathways; 15 are oxidoreductases, and only three fall within our definition (9%; Table 1).

**Figure 3:**
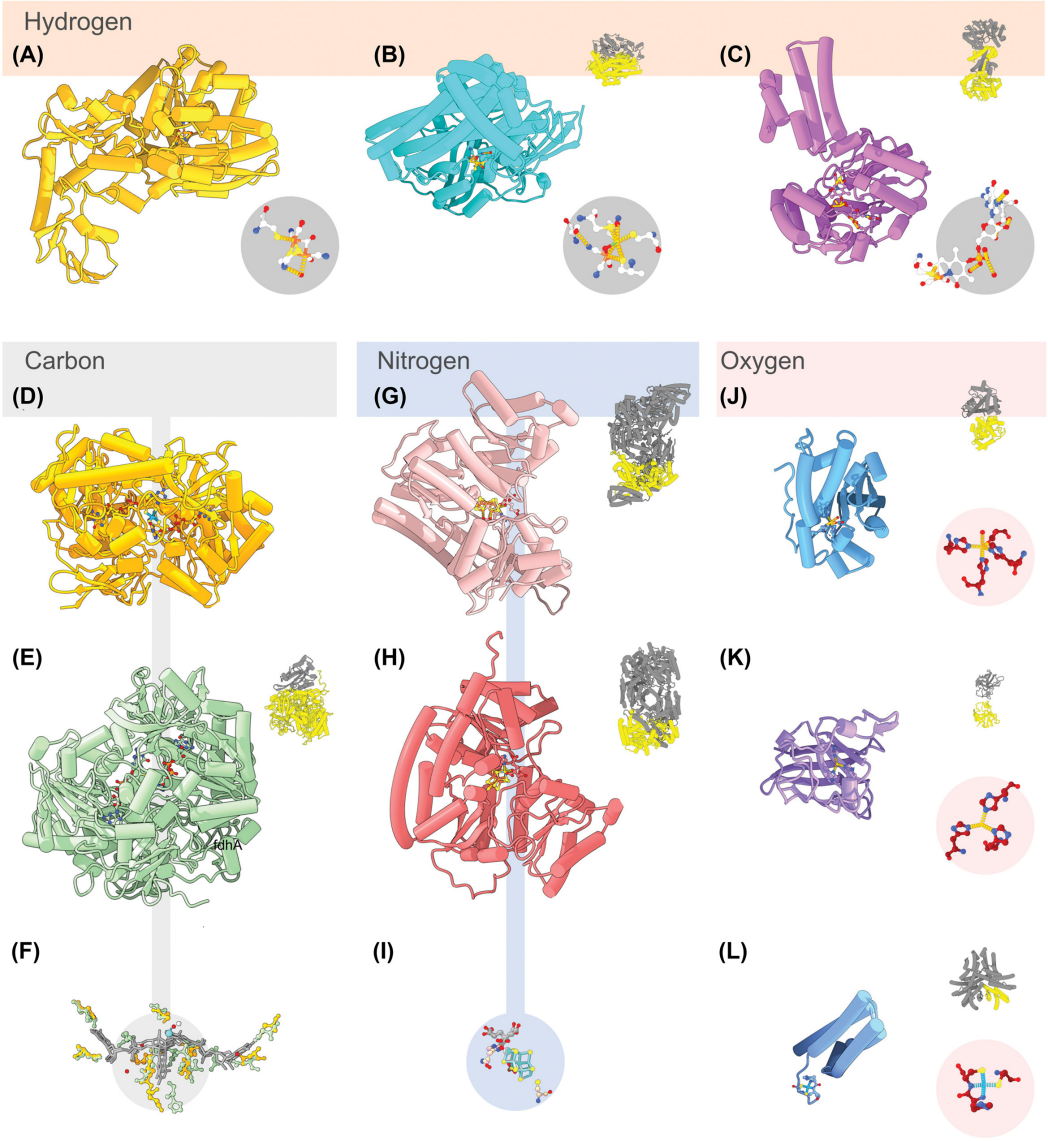
Structures of metal-containing subunits associated with prokaryotic metalloproteins relevant for biogeochemical cycles. The structures reported are relative to the hydrogen, carbon, nitrogen, and oxygen cycle and have been selected because they have isoforms containing different metals in their catalytic site or show some degree of cofactor cambialism. For each pdb, the coordinated metal/organometallic complex is displayed within a circle, together with a miniature of the assembly highlighting in yellow the catalytic subunit. Hydrogen cycle-related structure**s**: (**A**) FeFe-hydrogenase small subunit (*hydA*) from *Clostridium pasteurianum* (6N59); (**B**) NiFe-hydrogenase large subunit (*hydB*) from *C. pasteurianum* (5XLF; *hydA-hydB* heterodimeric assembly [AB]); (**C**) activated Fe-hydrogenase (*hmd*) from *Methanococcus aeolicus Nankai-3* (6HAV).** Carbon cycle-related structures:** (**D**) formate dehydrogenase H α-chain (*fdhF*) from *Escherichia coli* (1FDO); (**E**) Tungsten formylmethanofuran dehydrogenase chain α (*fwdA*) from *Methanothermobacter wolfeii* (5t5i, dodecameric assembly 2x[ABCDFG]); (**F**) the Mo/W-bis(molybdopterin guanine dinucleotide) cofactor common to both enzymes. Nitrogen cycle-related structures: (**G**) V containing nitrogenase α-chain (*vnfD*) from *Azotobacter vinelandii* (5N6Y; hexameric assembly 2x[ABC]); (**H**) Mo containing nitrogenase α-chain (*nifD*) from *A. vinelandii* (53U7Q; tetrameric assembly 2x[AC]); (**I**) the FeMoco/FeVco cofactor. Oxygen cycle-related structures**:** (**J**) Superoxide dismutase (*sodA*) from *Deinococcus radiodurans* (1Y67, 3KKY; homodimeric assembly); (**K**) Superoxide dismutase (*sodC*) from *Photobacterium leiognathi* (1BZ0; homodimeric assembly); (**L**) Ni-containing superoxide dismutase (*sodN*) from *Streptomyces selenosis* (1Q0G; hexameric assembly).

Consulting KEGG, we observed that the active centers of oxidoreductases in the seven carbon fixation pathways usually consist of organic cofactors such as ferredoxin, FAD, and NAD (see supplementary online materials). A few exceptions exist. *Escherichia coli* formate dehydrogenase (*fdhF* [1FDO], Figures 2A and 3D) and *Methanothermobacter wolfeii* formylmethanofuran dehydrogenase (*fwdA* [5T5I], Figure 3E and Supplementary Figure S2) are homologous enzymes involved in CO_2_ fixation. Their active site enzymes contain a molybdenum ion bound to two molybdopterin guanine dinucleotide (PDB accession MGD) and a selenocysteine or a tungsten ion bound to two MGD and a cysteine, respectively.

The carbon monoxide dehydrogenase (*coxL* [1ZXI], Figure 2C) is also a metal-containing oxidoreductase of biogeochemical interest. It uses either a Cu-S-Mo cluster for the aerobic variant or a Cu-Ni or Ni-only cofactor for the anaerobic variant of the enzyme (*codh* [1MGJ], Figure 2D) [16,17]. The Cu-S-Mo cluster associated with the aerobic CODH interacts with a single molybdopterin cytosine dinucleotide (MCN) rather than two (as in the FDH). The two oxygens of the cluster replace the dithiolate group of the second MCN in defining the metal geometry (here constrained to be distorted pyramidal) [18]. For anaerobic CODH, Ni is integrated within a Fe-[NiFe_3_S_4_] cluster rather than being bridged to a cubane [Fe_4_S_4_] [19].

Within the methane cycle, two additional enzymes match our definition of biogeochemically relevant metal oxidoreductase: the membrane-bound particulate Methane monooxygenase (pMMO, *pmoB1/B2* [3RGB]), which uses Cu as a catalytic cofactor, and the cytoplasmic, copper starvation-induced soluble Methane monooxygenase (sMMO, *mmoX* [1MHY], Figure 2B), which in turn uses Fe-Fe. Albeit both catalyze methane oxidation, they are entirely different from a structural standpoint.

**Figure 4:**
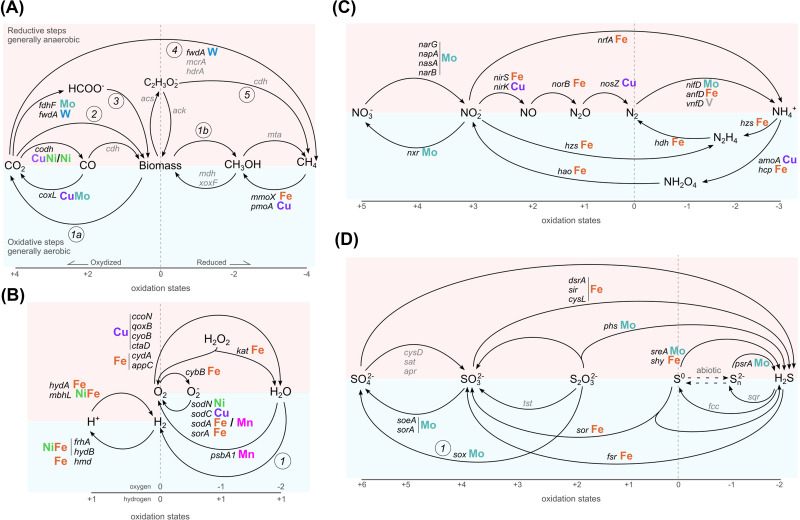
Biogeochemical cycles of the major CHNOS elements. The reductive side of each cycle is reported on the upper side of each cycle and highlighted in light red, while the oxidative side is on the bottom and highlighted in light blue. Molecules in each element are ordered left to right based on the oxidation state starting with the most oxidized form. Key enzymes for each step of each cycle are reported: in black the names of enzymes that do not meet our criteria of biogeochemically relevant metal containing oxidoreductases, while in light gray other enzymes. Enzyme names are based on the KEGG names and reported in Supplementary Table S1. The catalytic metal is reported for each biogeochemical oxidoreductase colored according to Jmol color scheme. The numbers in a circle represent complex pathways/processes. (**A**) Carbon cycle: 1a and 1b, heterotrophy and fermentation: no enzyme meets our criteria in these pathways; 2, carbon fixation; 3, formate assimilation; 4, methanogenesis pathway: the reported enzyme catalyze key steps in this multi-enzyme pathway; 5, acetoclastic methanogenesis. (**B**) Hydrogen and oxygen cycle: 1, abiotic and biotic hydrogen formation; note that the oxidation scale for oxygen and hydrogen are distinct, and hydrogen is reported with the same oxidation state on both sides. (**C**) Nitrogen cycle. (**D**) Sulfur cycle: 1, sulfur/thiosulfate oxidation is accomplished by a complex group of enzymes (*sox*) of which *soxCD* meets our criteria (Table 1).

Despite the low number of biogeochemical metal-containing oxidoreductases present, the carbon cycle is very diverse in its metal requirement, with Fe, Mo, W, Cu, and Ni involved in key steps of the cycle (Figure 4A).

## Nitrogen cycle

Nitrogen is abundant in earth’s atmosphere in the form of dinitrogen (N_2_) gas and it is present in significant quantities also in the mantle. This element is vital in building nucleic acids, proteins, and enzymes. At the enzymatic level, nitrogen can be transformed between different compounds with different redox states, e.g*.*, NH_4_^+^, NO, NO_2_, N_2_O, NO_3_^−^, NO_2_^−^, hydroxylamine, and amino acids, moving from +5 in NO_3_^−^ to -3 in ammonia [20] (Figure 4C). The 22 different EC numbers present in the energetic nitrogen cycle on KEGG are oxidoreductases, and 16 (73%) of them are metalloenzymes relevant in our context. The most frequent metal is Fe, followed by Mo, Cu, and V (Table 1).

Nitrification is governed by Fe, except for the Cu-containing cofactor known as cupredoxin [21–23]. The most utilized metal cofactor for denitrification involves Mo, followed by Fe and Cu, with different geometry inside the enzymatic cofactors. The dissimilatory nitrite reduction and the assimilatory nitrate/nitrite reduction are controlled by Fe, except for the nitrate reductases, in which the catalytic metal is Mo [24]. The anaerobic oxidation of ammonia is carried out by Fe-containing enzymes (Figure 4C). The nitrogen fixation pathway is carried out by the Nitrogenase enzyme (Figures 2F and 3G,H), which exists in three different isoforms partnering with a unique cofactor: FeMoco (*nifD* [3U7Q], Figure 3H), FeVco (*vnfD* [5N6Y], Figures 2I and 3G,I and Supplementary Figure S3), or FeFeco [25]. Fe is the leading metal in every step of the nitrogen cycle associated with the more reduced nitrogen molecules. In contrast, Mo and Cu are associated with the most oxidized forms of nitrogen or enzymes directly involving molecular oxygen (e.g*.*, Ammonia monooxygenase) (Figure 4C).

## Sulfur cycle

Sulfur is the 10th most abundant element on Earth. Despite only a small fraction of it being bound to biomass, it is essential in all organisms. Life plays key roles in the global sulfur cycle through its assimilation into methionine and cysteine, enzyme cofactors (i.e*.*, iron-sulfur clusters), and through its use as electron donor/acceptor in dissimilatory energy-yielding reactions (mainly restricted to prokaryotes) [26]. The sulfur cycle involves reactions between eight valence states, from the most reduced H_2_S (−2) to the most oxidized SO_4_^2−^ (+6, Figure 4D). Among the 19 enzymes involved in the cycle, 14 are oxidoreductases, and 11 of these fall within our definition (79% of all sulfur cycle enzymatic classes), relying on the presence of either Mo or Fe for their catalytic activity and having a direct biogeochemical impact through their function (Table 1).

The aerobic sulfur disproportionation, assimilatory sulfate reduction, and sulfate reduction pathways are catalyzed by Fe-containing enzymes (Table 1 and Figure 4D). DMSO reduction, sulfite oxidation, sulfur disproportionation, and thiosulfate reduction pathways are catalyzed by Mo-containing enzymes. Interestingly, both dimethyl sulfide:cytochrome c2 reductase (DMSO reduction) and sulfite dehydrogenase (sulfite oxidation) contain a molybdenum-bis (molybdopterin guanine dinucleotide) geometry. Additionally, some pathways of the sulfur cycle involve steps catalyzed by enzymes containing both Fe and Mo. For instance, sulfhydrogenase (Fe-containing, part of a NiFe hydrogenase multienzyme complex) and sulfur reductase (Mo-containing) can catalyze sulfur reduction [27]. The same pattern is observed in the thiosulfate oxidation pathway, with sulfane dehydrogenase (Mo-containing) and Thiosulfate dehydrogenase (Fe-containing). This difference could be due to the different substrates these enzymes interact with, as sulfhydrogenase interacts with hydrogen and sulfur reductase with oxygen, suggesting that the redox potential of these substrates could provide selective pressures for specific metal utilization.

## Oxygen cycle

The great availability of oxygen in earth’s extant atmosphere results from the emergence of oxygenic photosynthesis, which, coupled with a complex series of geological feedbacks, was responsible for the Great Oxidation Event (GOE, 2.5–2.3 billion years ago) [28,29]. Photosystem II (PS-II) is the main protein complex involved in oxygenic photosynthesis. The oxygen-evolving complex (OEC) represents the PS-II catalytic site where the manganese-dependent photo-oxidation of water occurs, with subsequent release of oxygen (Figure 4B) [30,31]. The presence of Mn ions in the OEC catalytic center is supposedly a consequence of its abundance in the Archean oceans and its hypothetical former use as a phototrophic electron donor [32–34]. Furthermore, enhanced oxygen availability prompted the evolution of both O_2_-respiratory and -detoxifying mechanisms [35].

Oxygen high electronegativity makes it a suitable terminal acceptor in oxidative phosphorylation, the hallmark of aerobic respiration, where oxygen reduction to water is carried out by cytochrome oxidases (classified as translocases, EC 7.1) (Figure 4B) [36–38]. These enzymes generally require Cu as a metal cofactor, directly located in the catalytic center and coordinated by a heme group. Cytochrome *bd* ubiquinol oxidases make an exception, as their only metal cofactor is Fe, complexed in a heme group (*cydA, appC* [6RKO, 7OY2] Figure 2L; Table 1) [31,38–42]. In *E. coli*, Cu-containing cytochrome *bo* is maximally synthesized under high oxygen availability. Conversely, iron-containing cytochromes *bd* predominate in microaerophilic conditions [43], showing a very low *K*_m_ for oxygen and a less efficient proton motive force [44,45]. This evidence suggests that the nature of the metal cofactor is crucial in determining cytochromes’ performance and their affinity for oxygen.

On the other hand, aerobic respiration induces the formation of reactive species of oxygen (ROS) (Figure 4B), which are responsible for cell damage [46]. Superoxide radical anions can be detoxified by three superoxide dismutase (SOD) families, which differ in the catalytic metal (*e.g.*, Fe/Mn, Cu, and Ni) (*sodA, sodC, sodN* [1Y67/3KKY, 1BZ0, 1Q0G] Figure 3J,K,L; Table 1) [47–49]. Among them, the Fe/Mn family is highly flexible in cofactor utilization, representing a clear example of a cambialistic enzyme (*sodA/B* [1y67/3kky], Supplementary Figure S2) [48,50]. Hydrogen peroxide produced by SODs is rapidly detoxified by the catalase-peroxidase, whose metal cofactor is Fe in a heme conformation (Figure 4B) [51].

## Hydrogen cycle

Hydrogen is a key reduced compound in the redox balance of the planet. It is produced by several abiotic processes, including water photolysis/radiolysis, hydrothermal reactions, magmatic degassing, and hydration of iron-rich ultramafic rocks [52]. Hydrogen is also produced and consumed by microorganisms and used as an electron donor—it is one of the main energetic currencies exchanged within microbial communities [53]. Microorganisms can interact with molecular hydrogen through a group of diverse enzymes called hydrogenases, which catalyze the conversion of molecular hydrogen to protons and electrons and H_2_ regeneration through the reverse reaction [54,55] (Figure 4B). Their specialized metallic centers coordinate dihydrogen, polarizing the molecule to induce its heterolytic splitting into a proton and a hydride ion.

There are three main groups of hydrogenases, NiFe containing hydrogenases (*hydA* [6N59] Figures 2E and 3A), FeFe hydrogenases (*hydB* [5XLF] Figures 2G and 3B), and Fe-only hydrogenases (*hmD* [6HAV] Figure 3C). [NiFe]-hydrogenases are found in many Bacteria and Archaea, [FeFe]-hydrogenases in Bacteria and some eukaryotes, and [Fe]-hydrogenases only in Archaea [53]. Of the 30+ classes of hydrogenases known, we report here an example of H_2_-consuming and H_2_-producing hydrogenases from the main [NiFe], [FeFe], and [Fe] hydrogenases (Table 1 and Figure 4). [NiFe]-hydrogenases are mainly involved in H_2_ oxidation but have many other functions such as H_2_ evolution, sensing, CO respiration, electron bifurcation, and cofactors reduction [53,56,57]. In selenium-rich conditions, some Bacteria, like *Desulfovibrio vulgaris*, downregulate the production of [NiFe]-hydrogenases in favor of protein variants with selenocysteine as one of Ni ligands, displaying lower inhibition by molecular hydrogen and lower O_2_ sensitivity [58–60]. [FeFe]-hydrogenases also serve diverse physiological functions such as H_2_ uptake, sensing, evolution, electron bifurcation, and CO_2_ fixation [61]. [Fe]-hydrogenases, the least characterized type of hydrogenases, have only been detected in methanogenic Archaea where they are expressed when Ni is limiting [62,63].

## Cofactor cambialism at the core of biogeochemistry

Diverse factors constrain the choice of metals at the core of metabolisms: the environmental availability of the element of interest, its suitability for the specific redox reaction to be catalyzed, and the ability to control its binding to the target enzyme. Theoretically, metal-binding affinities of natural proteins are defined by the ligand field stabilization energy of metal ions and follow the Irving–Williams (IW) series (Mn^2+^ < Fe^2+^ < Co^2+^ < Ni^2+^ < Cu^2+^ > Zn^2+^) [64]. In practice, cells tend to maintain the availability of metal ions inverse to the IW series [65] so that binding is more regulated by ion availability in the immediate environment of the metalloprotein (or the metallochaperone), with very high spatial granularity-cells are not ideal solutions. Moreover, ions' concentration can change to the point that different metals can be acquired when folding in different places [66]. The environmental concentration is modulated by metal transport and the metal bioavailability in the outer environment—computational studies have shown that if one removes metallochaperones, metal specificity becomes strongly correlated with metal abundance in the environment. The situation is further complicated because cations have overlapping characteristics that impede absolute specificity. For some metals, similarity in binding affinity and preference over coordination environments is associated with different redox chemistry (e.g*.*, Mn^2+^/Mg^2+^/Fe^2+^ and Mo/W). In this context, excluding the wrong metals from proteins may be more challenging than acquiring the right ones [67], and having a metallochaperone or an additional metal center (as in binuclear Mg^2+^, [68] could reduce mismetallation.

At the environmental level, the (bio)availability of metals might control to a first order its utilization by biology [11]. However, the metal used also depends on the enzyme's evolutionary trajectory. The idea that ancient, promiscuous oxidoreductases were constrained to use bioavailable metals to catalyze redox reactions and that a contingency shaped evolution of more ‘focused’ metalloenzymes differing in metal utilization is supported by comparison of proteomes across life domains [69]. It is worth remembering that many of the transition metals detailed in this review were readily available in ancient times due to the low oxygen/high sulfur environment, except for Cu, Mo, and Zn (that are sparingly soluble in those conditions) and that the Paleozoic oxidation event (GOE) reverted this trend [70]. At the same time, it is essential to consider that selection ‘locked in’ some crucial enzymes (*e.g.*, Fe-S proteins, [71] relying on once-plentiful metal species (after the GOE, iron is primarily available in the low-solubility ferric form).

Currently, biogeochemical cycles are dominated by Fe as a key catalytic metal (Figure 4 and Supplementary Figure S4). However, its ability to interact with oxidized substrates is often limited to low-concentration conditions requiring high affinity (like in the Fe-containing cytochromes used under microaerophilic conditions). As a result, cells rely on Cu and Mo to attain the higher redox potential needed to interact with powerful oxidants-such as oxygen in full aerobic conditions, nitrate, and other oxidized nitrogen species. Determining the *in vivo* utilization of metal ions by biomolecules is challenging since complicated metal centers can remain poorly defined even after structure determination due to, *e.g.*, experimental procedure-related substitutions.

## Conclusion

Life sustains itself through redox reactions that capitalize on environmental thermodynamic disequilibria. A reasonable hypothesis is that life became proficient in redox reactions as it evolved. In prebiotic Earth, metal ions alone were sufficient redox agents. As the concentration of organic molecules in the environment increased, organometallic complexes formed. The existence of these complexes, in turn, created the context for metallopeptides evolutions that eventually developed into metalloproteins [72]. Extant life is capable of catalyzing a large number of redox reactions, despite this a quantitative understanding of the diversity and distribution of thermodynamic plausible (i.e*.*, energy yielding) reactions are lacking, and a number of theoretically possible reactions have yet to be identified in nature [73].

The requirement of life for metals as cofactors in key biogeochemical reactions attests to the vital role that metals play in the functioning of Earth and the intricate relationship between the biosphere and the geosphere. Complex stellar processes, protoplanetary disk accretion, and planetary differentiation [74], changing redox conditions during planetary evolution [75,76], plate tectonics, supercontinent assembly [77], and changes in dominant volcanism [78], all contributed to the complex interactions between metal bioavailability and the evolution of biogeochemistry. Nevertheless, our understanding of the role of metals in controlling microbial metabolism and biogeochemistry is still in its infancy. Critical questions about selective pressures imposed by redox potentials of substrates and reaction products in selecting specific metals and the effect of metal environmental availability remain open. In addition, we still need a complete catalog of the elements life uses in protein structure; the diversity of organometallic structures has been poorly examined in environmental—and mostly unculturable [79]—microbes, making it problematic to investigate protein structures and cofactors using traditional approaches. Increasing our knowledge of organometallic cofactors from uncultured microbial groups can revolutionize our understanding of how redox chemistry mediates the interaction between life and our planet, offering promising possibilities in the green chemistry industry and opening our transition to a more sustainable economy [80–82].

## Summary

- There is a universal need for redox chemistry by life to use thermodynamic disequilibrium.
- Biogeochemical cycles, and therefore the functioning of our planet, are controlled by a small number of biogeochemically relevant redox proteins, most of which use metal cofactors. The metal used is generally tuned together with the protein structure to the midpoint potential of the reaction catalyzed, although alternatives are possible.
- Metal choice is dictated on the first order by availability and active transport and refined by protein structure. Besides, evolution contributed to ‘frozen accidents’ that irreversibly paired some metals to specific cycles.
- The correlation between the diversity of metal cofactors and the biogeochemical redox reactions in which they are involved is still unclarified.
- Despite their importance for our planet’s functioning, we have limited information regarding the organometallic structure found in oxidoreductases of uncultured lineages of microorganisms.

## Supplementary Material

Supplementary Figures S1-S4 and Table S1

## Abbreviations

GOE: Great Oxidation Event
MCN: molybdopterin cytosine dinucleotide
OEC: oxygen-evolving complex
ROS: reactive species of oxygen
SOD: superoxide dismutase
