# Supplementary material for: Oxidoreductases and metal cofactors in the functioning of the earth
Source: Essays Biochem. 2023 Aug 11;67(4):653–70. doi: 10.1042/EBC20230012 (PMC10423856; doi:10.1042/EBC20230012)
Supplement: Supplementary Figures S1-S4 and Table S1 [file EBC-2023-0012C_supp.pdf]

Oxidoreductases and metal cofactors in the functioning of Earth

Bruno Hay Mele[1], Maria Monticelli[2,1], Serena Leone[3], Deborah Bastoni[1], Bernardo Barosa[1], Martina Cascone[1], Flavia Migliaccio[1], Francesco Montemagno[1], Annarita Ricciardelli[1], Luca Tonietti[1,4], Alessandra Rotundi[4], Angelina Cordone[1], Donato Giovannelli[1,4,5,6,7,8]

1 Department of Biology, University of Naples Federico II, Naples, Italy
2 National Research Council – Institute of Biomolecular Chemistry - CNR-ICB, Pozzuoli, Italy
3 Dipartimento di Biologia ed Evoluzione degli Organismi Marini, Stazione Zoologica Anton. Dohrn, Napoli, Italy
4 Department of Science and Technology, University of Naples Parthenope, Naples, Italy
5 National Research Council – Institute of Marine Biological Resources and Biotechnologies - CNR-IRBIM, Ancona, Italy
6 Department of Marine and Coastal Science, Rutgers University, New Brunswick, NJ, USA
7 Marine Chemistry & Geochemistry Department - Woods Hole Oceanographic Institution, MA, USA
8 Earth-Life Science Institute, Tokyo Institute of Technology, Tokyo, Japan

Corresponding authors: Bruno Hay Mele (bruno.haymele@unina.it) and Donato Giovannelli (donato.giovannelli@unina.it)

Supplementary Online Materials

Supplementary Methods

The enzymatic classes involved in the energetic pathways of the different biogeochemical cycles were counted from KEGG, querying the KEGG REST API service for cross-references between pathway (map entries) or modules (Module entries) and enzyme classes. The service deduplicates repeated enzymatic classes. Enzymatic classes not cross-referenced by the service were manually added. These are:

- EC:1.7.1.10, EC:1.7.2.7, EC:1.7.2.8 for nitrogen cycle, as they are involved in the anammox pathway;
- EC:1.2.5.3 for carbon cycle, as it is essential for aerobic oxidation of CO to CO_2;
- EC:1.13.11.18, EC:1.13.11.55, EC:1.8.2.1, EC:1.8.5.5, EC:1.8.5.6, EC:1.97.1.3, EC:1.12.98.4, as they are crucial for energetic sulfur metabolism.

Supplementary Table 1. Enzyme names used in Figure 4.

Enzyme abbreviation	Enzyme name
fdhF	Formate dehydrogenase
fwdA	Tungsten formylmethanofuran dehydrogenase
codh	Anaerobic carbon monoxide dehydrogenase
coxL	Aerobic carbon monoxide dehydrogenase
cdh	acetyl-CoA decarbonylase/synthase
acs	Acetate synthase
ack	Acetate kinase
mcrA	Methyl-coenzyme M reductase
hdrA	Heterodisulfide reductase
mta	Methyl-Co(III) coenzyme M methyltransferase
mmoX	Soluble methane monooxygenase
pmoA	Particulate methane monooxygenase
mdh	Malate dehydrogenase
xoxF	Lanthanide-dependent methanol dehydrogenase
hydA	(FeFe) Iron hydrogenase 1
mbhL	(NiFe) Membrane-bound hydrogenase
frhA	(NiFe) F420-reducing hydrogenase
hydB	Periplasmic [NiFe] hydrogenase
hmd	5,10-methenyltetrahydromethanopterin hydrogenase
ccoN	Cbb3-type cytochrome c oxidase
qoxB	Cytochrome ba quinol oxidase
cyoB	Cytochrome bo(3) ubiquinol oxidase
ctaD	Cytochrome-c oxidase polypeptide I + III
cydA	Cytochrome bd-I ubiquinol oxidase
appC	Putative cytochrome bd-II ubiquinol oxidase
kat	Catalase-peroxidase
cybB	Superoxide oxidase
sodN	Superoxide dismutase [Ni]
sodC	Superoxide dismutase [Cu-Zn]
sodA	Superoxide dismutase [Mn]
sorA	Superoxide reductase
psbA1	Photosystem II protein D1 1
narG	Respiratory nitrate reductase 1
napA	Periplasmic nitrate reductase
nasA	Nitrate reductase [NADPH]
narB	Nitrate reductase
nxr	Nitrite oxidoreductase

nrfA	Cytochrome c nitrite reductase
nirS	Nitrite reductase
nirK	Copper-containing nitrite reductase
norB	Nitric oxide reductase
nosZ	Nitrous-oxide reductase
nifD	Nitrogenase molybdenum-iron protein
anfD	Nitrogenase iron-iron protein
vnfD	Nitrogenase vanadium-iron protein
hsz	Hydrazine synthase
hdh	Hydrazine dehydrogenase
amoA	(Cupredoxin) ammonia monooxygenase
hcp	Hydroxylamine reductase
hao	Hydroxylamine oxidoreductase
cysD	Sulfite reductase [NADPH] hemoprotein
sat	Sulfate adenylyltransferase
apr	Assimilatory adenylylsulfate reductase
soeA	Sulfite dehydrogenase
sorA	Superoxide reductase
sox	Sulfur-oxidation complex
tst	Thiosulfate sulfurtransferase
dsrA	Sulfite reductase, dissimilatory-type
sir	Assimilatory sulfite reductase (ferredoxin)
cysL	Sulfite reductase [NADPH] hemoprotein
phs/psrA	Polysulfide reductase
sreA	Sulfur reductase
shy	Sulfhydrogenase
sqr	Sulfide:quinone oxidoreductase
fcc	Sulfide dehydrogenase
fsr	Coenzyme F420-dependent sulfite reductase
sor	Sulfite:cytochrome c oxidoreductase

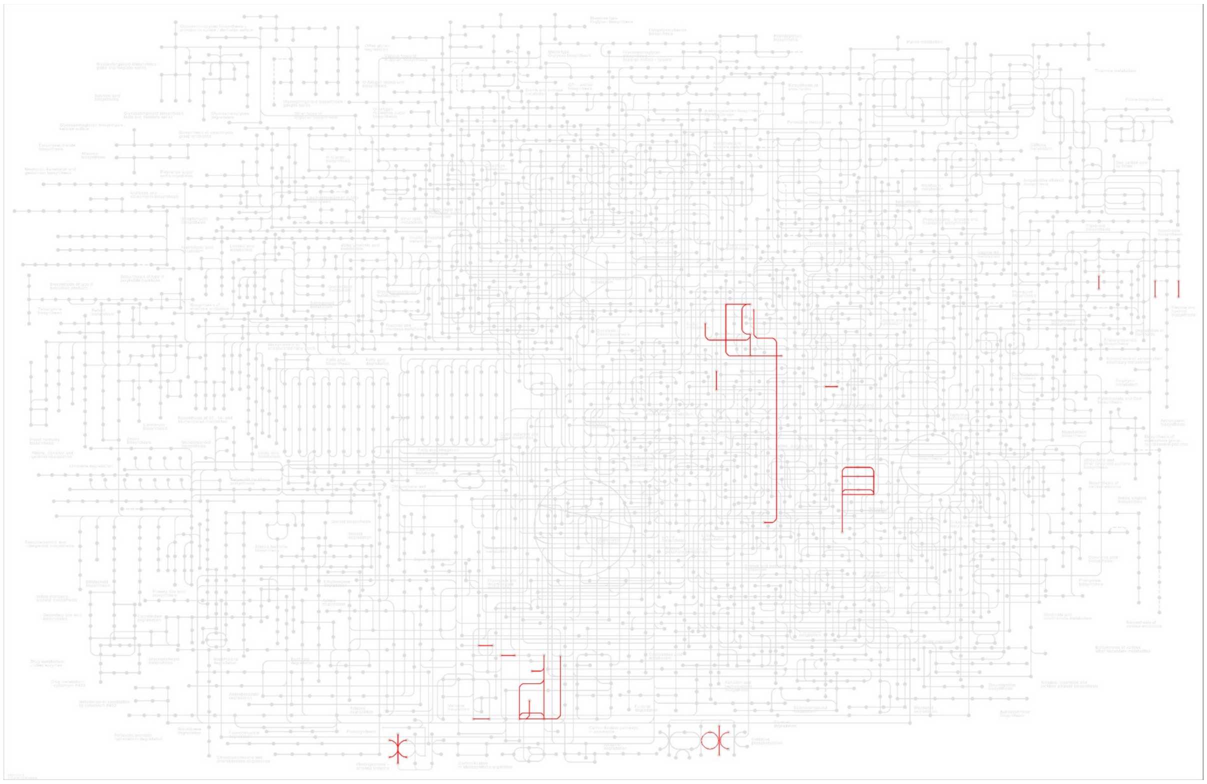

Supplementary Figure S1. Biogeochemically-relevant oxidoreductases as defined in this work highlighted in the KEGG metabolic map (PATH:MAP0100).

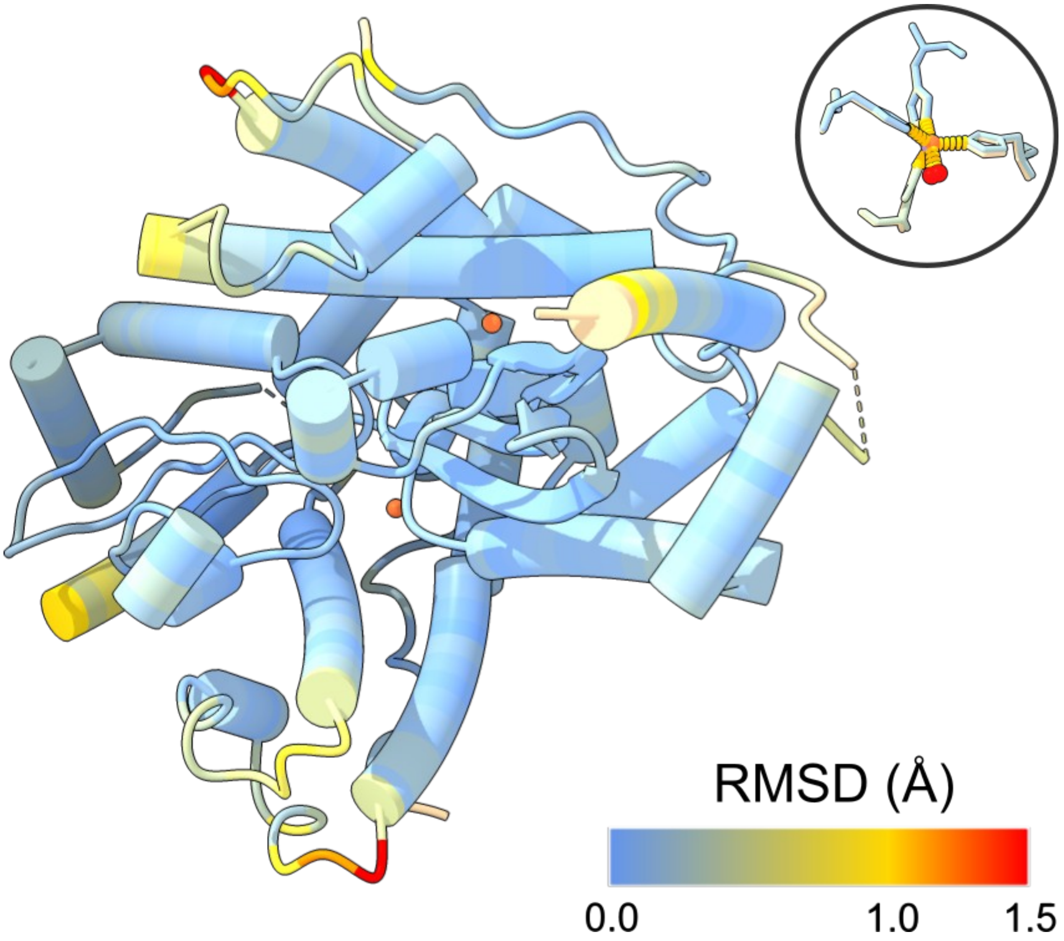

Supplementary Figure S2. Structural alignment of cambialistic [Fe-Mg] Superoxide dismutase gene (*sodA*) from *Deinococcus radiodurans* (1Y67, 3KKY; homodimeric assembly). <RMSD> = 0.277, $RMSD_{residue}$ as per color key, using 1Y67 as reference. Inset: detail of the inner coordination sphere for the two metals.

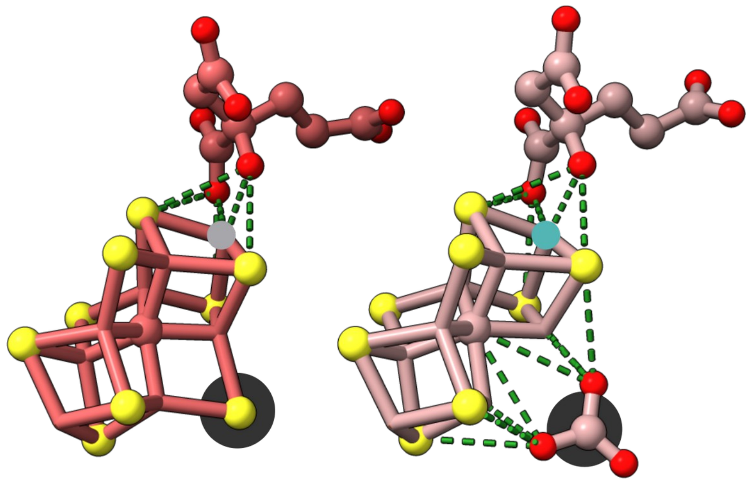

(A) (B)

Supplementary Figure S3. Details of *vnfD*-associated FeVco (A) and *nifD*-associated FeMoco (B). Vanadium and molybdenum are filled disks (JMol color code); positional equivalents (Fe in FeVco and CO3 in FeMoco) are highlighted by dark gray disk.

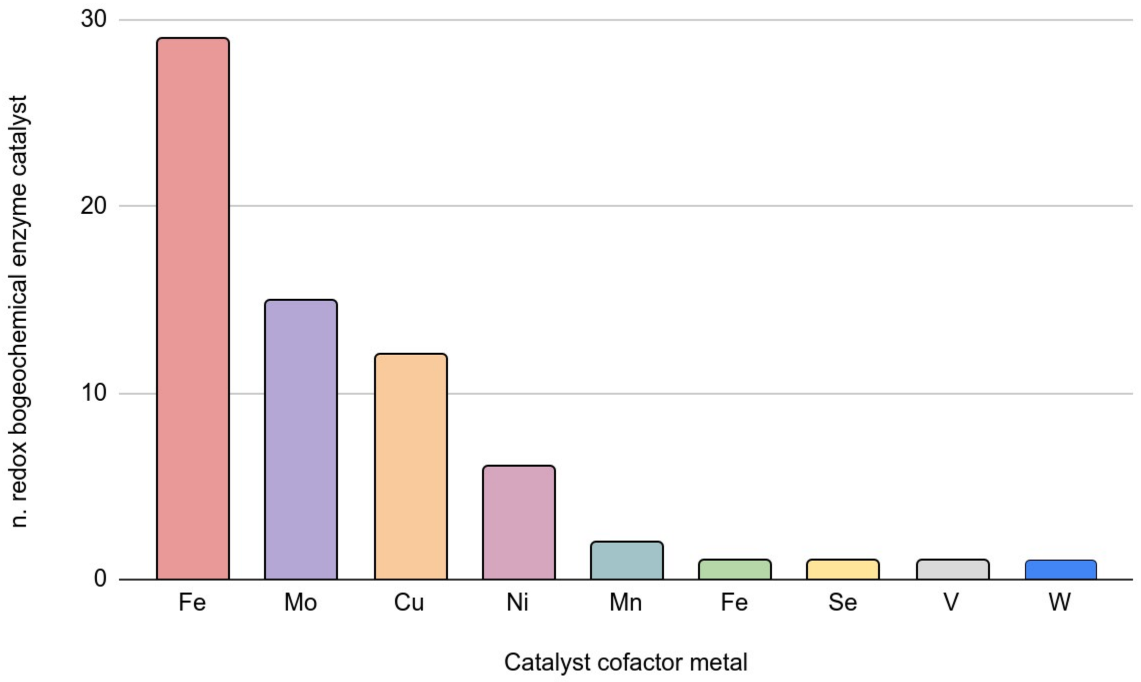

Supplementary Figure S4. Number of metals found in biogeochemically-relevant oxidoreductases as defined in this work.
